# On the Prevention of Pitting in Smallpox

**Published:** 1856-04

**Authors:** James Wallace


### *On the Prevention of Pitting in Smallpox.* By Dr. JAMES WALLACE.

(Glasgow Medical Journal, July, 1855.)

After trying several means for preventing pitting in smallpox, Dr. Wallace arrives at the conclusion that gutta-percha dissolved in chloroform (as originally suggested by Dr. Stokes,) is more effectual than any other; but that even this will not entirely prevent the deformity. He finds, also, that it is sufficient to make the application just before maturation is complete.

"In 1849, when acting as clinicle clerk in the Glasgow Royal Infirmary, I painted with collodion—as originally suggested by Dr. Ranking, in the Lancet for January of that year—one half of the face of a patient laboring under semi-confluent smallpox, and was so much struck with its efficacy in mitigating disfigurement, that, on taking charge of the Greenock Infirmary, I determined on giving it a farther trial. I employed it, accordingly, in eleven cases, coming under my care in the spring and winter of 1852. Of these, five were confluent, two semi-confluent, three with the eruption discrete and moderately copious, and one with the eruption discrete, but very abundant. In one, the collodion was applied on the first day of the eruption; in another, on the second; in five, on the third; and in four, on the fourth. In all of them, it was used some days previous to the stage of maturation, and the result in general was satisfactory, the pitting having been prevented to a considerable extent. But during the progress of the disease, the application was attended with no small inconvenience; for, when tumefaction set in, the collodion, from its want of elasticity, kept the parts in such a state of pain, heat and tension, that several of the patients were with difficulty persuaded to let it remain on. For the same reason, also, it cracked so frequently, that gaps, which were formed here and there, had to be filled up by a reapplication of the solution, which, on subsidence of the swelling, became still farther necessary, because of the less prominent parts of the face being separated from the artificial pellicle. Nor were these disadvantages compensated for by anything like what has been called *abortion* of the pustules, for the progress of the eruption was just as if the face had been without any protection whatever, the only beneficial result having been mitigation of the pitting. Now, as this was the principal object in view, it became evident that the same effect was as likely to be produced, and with much less uneasiness to the patient, by having recourse to the application at a later stage of the disease; and as the efficacy of such a covering could depend, from what was observed, only on the exclusion of atmospheric air from the pustules when fully developed, and on its allowing cicatrization to advance in a way analogous to what is termed the modelling process,* the most suitable period seemed to be that immediately before complete maturation, when there is usually found a greater or less amount of swelling. By this method the face, on any subsequent increase in size, would be subject to considerably less constriction than by that first followed; and, on falling to its normal dimensions, would have the coating adherent to a much greater extent. But there would still be a greater drawback in the inelasticity of the collodion, as well as in its liability to crack. I therefore gave up the use of that substance altogether, and had recourse to a solution of guttapercha in chloroform,† which I have now employed in twenty cases,‡ four being confluent, seven semi-confluent, seven with the eruption discrete and moderate, and two with the eruption discrete but copious. In the whole

* Miller's Principles of Surgery, p. 199.

† This seems to have been first used in cases of smallpox by Dr. Stokes of Dublin. Vide paper in "Dublin Quarterly Journal of Medical Science," for August, 1852, by the late Dr. Graves. According to the latter observer, it should not be employed till the eruption is fully matured, or even begins to exhibit the first appearance of collapse; but this stage is highly objectionable, in consequence of the cuticular covering of the pustules being then so much thinned, as to break readily while the solution is being applied.

‡ The whole of these, as well as those treated with collodion, are exclusive of fatal cases.

of them it was painted on immediately before complete maturation, which occurred in three on the fifth day of the eruption, in three on the sixth, in ten on the seventh, in two on the eighth, in one on the ninth, and in one on the tenth. Under this plan, the increase that took place in the swelling was found to be in general moderate, and not such as to prevent the gutta percha yielding readily to it, nor to cause that feeling of heat and tension which was so much complained of by the patients subjected to the collodion application; and when the tumefaction subsided, the mask was found to adhere intimately to all the subjacent parts, except at the angles of the mouth, where, from the frequent motion of the lips, it invariably became detached. But this was easily remedied, either by touching lightly with a camel-hair pencil dipped in chloroform, the loose portion of the covering, which in that way became softened and collapsed upon the skin; or, which is better, by painting anew the exposed part of the face, without disturbing the separated pellicle at all. Farther than this no interference was necessary, the whole coating having been allowed to remain on till desquamation spontaneously occurred, when the face was found, in all the cases, to be in a very satisfactory condition. The ultimate result, too, was exceedingly favorable, scarcely any pitting having been noticed when the patients were dismissed, except in two or three instances, in which, however, it was moderate, and in which, but for the covering, there would, I am convinced, have been frightful disfiguration. At the same time it ought to be stated, that those cases which I had an opportunity of seeing, some months after their discharge, presented the marks much more distinctly than when under observation in the hospital, but still so modified, as to show that they had been very materially benefited by the means resorted to.

"From this it will be seen, that the gutta-percha, like the collodion, though to a much greater degree, succeeded only in mitigating, and not entirely preventing, pitting. Hence, some may be apt to conclude that its efficacy was much inferior to that of other applications, which have of late years been brought under the notice of the profession. But in estimating the comparative merits of these, regard should be had to the appearance of the patients, as exhibited long after they had been under treatment, as well as on their leaving the hands of the physician."

[*Ranking's Abstract.*

